# Chain Dynamics of Partially Disentangled UHMWPE around Melting Point Characterized by ^1^H Low-Field Solid-State NMR

**DOI:** 10.3390/polym15081910

**Published:** 2023-04-16

**Authors:** Yan Zhao, Yuling Liang, Yingjie Yao, Hao Wang, Tong Lin, Yun Gao, Xiaoliang Wang, Gi Xue

**Affiliations:** 1 Key Laboratory of High-Performance Polymer Materials and Technology of Ministry of Education, Department of Polymer Science and Engineering, School of Chemistry and Chemical Engineering, Nanjing University, Nanjing 210023, China; mg20240136@smail.nju.edu.cn (Y.Z.); 191850227@smail.nju.edu.cn (Y.Y.); lintong0124@163.com (T.L.); p709@nju.edu.cn (Y.G.); xuegi@nju.edu.cn (G.X.); 2 South China Advanced Institute for Soft Matter Science and Technology, School of Emergent Soft Matter, South China University of Technology, Guangzhou 510640, China; liang_yuling@126.com (Y.L.); wanghao18206@gmail.com (H.W.)

**Keywords:** entanglement, UHMWPE, solid-state NMR, crystallization, relaxation

## Abstract

Melts of ultrahigh molecular weight polyethylene (UHMWPE) entangled significantly, suffering processing difficulty. In this work, we prepared partially disentangled UHMWPE by freeze-extracting, exploring the corresponding enchantment of chain mobility. Fully refocused ^1^H free induction decay (FID) was used to capture the difference in chain segmental mobility during the melting of UHMWPE with different degrees of entanglement by low-field solid-state NMR. The longer the polyethylene (PE) chain is in a less-entangled state, the harder the process of merging into mobile parts after detaching from crystalline lamella during melting. ^1^H double quantum (DQ) NMR was further used to obtain information caused by residual dipolar interaction. Before melting, the DQ peak appeared earlier in intramolecular-nucleated PE than in intermolecular-nucleated PE because of the strong constraints of crystals in the former one. During melting, less-entangled UHMWPE could keep disentangled while less-entangled high density polyethylene (HDPE) could not. Unfortunately, no noticeable difference was found in DQ experiments between PE melts with different degrees of entanglement after melting. It was ascribed to the small contribution of entanglements compared with total residual dipolar interaction in melts. Overall, less-entangled UHMWPE could reserve its disentangled state around the melting point long enough to achieve a better way of processing.

## 1. Introduction

Ultra-high molecular weight endowed polyethylene with some unique properties [1,2,3], making it widely used as high-strength fibers [4], battery diaphragms [5], artificial joints [6] and so on. However, melts of UHMWPE entangled significantly [7], making it nearly incapable of flowing before degradation. The tube model predicted a molecular weight scaling of the reptation time as *τ*_d_∼*M*_w_^3^ [8,9]. Polyolefins with a molecular weight over 10^6^ g·mol^−1^ bore a relaxation time of around 5 s. While such relaxation time of polyolefins around 10^4^ g mol^−1^ was only 5 × 10^−5^ s [10,11]. Therefore, suppressing the degree of entanglement provided an effective solution to the processing of UHMWPE, e.g., UHMWPE prepared by dissolution of less than 5 wt% of it in high boiling solvent (such as decalin) showed improved processability [12]; heterogeneous UHMWPE melts obtained by slow melting of controlled synthesized UHMWPE showed decreased melt viscosity and provided enhanced drawability on crystallization [13]. Studying the role of entanglement during the melting process of solution-crystallized UHMWPE helped us understand the origin of various properties in UHMWPE. However, the role of entanglement in UHMWPE subjected to a melting process was seldom studied.

Entanglement of the semi-crystalline polymer was usually observed in the amorphous component next to crystallization, in its melts or solution state [14]. On the other side, entanglement did not occur in the crystalline phase of semi-crystalline polymer because crystals repelled entanglement during crystallization [15]. Entanglement impeded the motion of molecular chain segments and influenced the crystallization, rheology, morphology, and mechanical properties of polymer melts [16,17]. Much research was conducted to show the difference in chain dynamics among PE with different crystallization types. Isothermal crystallization experiments of PE showed that spherulites grew faster when the polymer was less entangled because the transportation of macromolecules toward the crystallization front was easier [9,17]. The difference was reduced when the annealing time increased before crystallization due to entanglements reconstitution.

Solid-state NMR was an essential method for researching the chain dynamics of PE. As a crystal-mobile polymer, PE showed mobility in the crystalline state, and the crystal thickness resulted from reorganization processes related to intracrystalline chain dynamics [18,19,20]. Yao et al. used ^13^C exchange NMR to investigate partially disentangled PE, finding the exchange process of methylene units from the amorphous to the crystalline state was faster than melt-crystallized ones [21]. Rastogi et al. followed the chain diffusion process between the amorphous and the crystalline regions among fully drawn disentangled UHMWPE, drawn entangled UHMWPE and commercially solution-spun UHMWPE fiber with the help of ^13^C cross-polarization magic-angle spinning solid-state NMR. The minimum amount of the mobile amorphous component was found in disentangled ones [22]. Compared to complex ^13^C high-field NMR, ^1^H low-field NMR method was simple and cost-efficient. Therefore, it was widely used in detecting the chain dynamics of polymers. Bärenwald et al. used Magic Sandwich Echo (MSE) in ^1^H low-field NMR to obtain information on the rate of chain flipping in PE microcrystals [23]. Litvinov et al. analyzed the density of physical entanglement networks in the amorphous phase of HDPE by ^1^H NMR *T*_2_ relaxation experiments [24], providing a new way to quantify the density of polymer entanglement.

Our previous works found that less entanglement could be partially reserved by freeze-extracting polymer from its dilute solution [25,26,27]. Such partially disentangled polymer crystallized faster and showed stronger enthalpy relaxation. Herein this work, we attempted to explain the origin of the processability enhancement of UHMWPE by decreasing the degree of entanglement from the perspective of chain dynamics. We prepared partially disentangled UHMWPE by freeze-extracting from dilute decalin solution. We checked the morphology and crystallization of partially disentangled samples with scanning electron microscopy (SEM) and differential scanning calorimetry (DSC). With the help of ^1^H fully refocused FID in low-field solid-state NMR, we could capture the difference in chain dynamics during the melting of PE with different degrees of entanglement. DQ NMR method was applied to explore the residual dipolar coupling caused by entanglements. Finally, we proposed a mechanism to elucidate the role of entanglement on the chain relaxation behavior of UHMWPE around the melting point. 

## 2. Materials and Methods

### 2.1. Materials

PE1.1M (UHMWPE, *M*_w_~1101 kg/mol, PDI~10.02) and PE0.5M (HDPE, *M*_w_~544 kg/mol, PDI~16.76) were kindly supplied by Celanese Chemical Co., Ltd. (Irving, TX, USA). Their weight average molecular weight (*M*_w_) and polydispersity (PDI) were measured by gel permeation chromatography (GPC) with trichlorobenzene as a carrier-solvent at 150 °C. 

The desired amount of PE was dissolved in decalin at 135 °C. The corresponding homogenous solution was added quickly into acetone ten times at room temperature. After strong stirring for 24 h, the decalin was extracted from the precipitate by acetone. Then the acetone with decalin was removed, and an equal amount of fresh acetone was added. This process was repeated four times to ensure the decalin was clearly extracted and removed. The final as-prepared PE was dried under a vacuum to remove acetone. The freeze-extracting method partially retained the degree of entanglement in the original solution [25,26,27]. The PE with [*η*]c value less than 1 (PE1.1M—0.1 wt% and PE0.5M—0.1 wt%) was regarded as less-entangled, while [*η*]c larger than 10 (PE1.1M—5 wt% and PE0.5M—10 wt%) was regarded as well-entangled [28,29,30]. Their intrinsic viscosity ([*η*]) was measured by a Ubbelohde viscometer with decalin as solvent at 135 °C, and c was the concentration of decalin solution of PE. More detailed information on the samples is listed in Table S1 (see in Supplementary Materials).

### 2.2. SEM

SEM experiments were performed on Hitachi S-4800 (Hitachi, Tokyo, Japan) with an acceleration voltage of 5.0 kV, and all the samples were coated with a thin gold layer for SEM observation. 

### 2.3. DSC

DSC was performed with a DSC 1 instrument (Mettler Toledo Corp., Zürich, Switzerland) to measure the melting point and crystallinity of polymers. Polymers (ca. 4 mg) were heated from 25 °C to 180 °C at different rates of 10 °C · min^−1^, 1 °C · min^−1^ and 0.1 °C · min^−1^. The melting temperature was taken at the peak. The crystallinity (*X*_c_) was calculated by comparison with the heat of fusion of a perfectly crystalline PE, i.e., 289 J · g^−1^ [31].

### 2.4. Rheological Measurement

Rheology experiments were all carried out on a strain-controlled rheometer ARES-G2 (TA Instruments, New Castle, DE, USA). A 25 mm diameter parallel plate with a typical gap of around 1 mm was used in linear viscoelastic measurements. For rheological measurements, PE1.1M bulk and PE0.5M bulk with 1 wt% antioxidant 1010 were hot pressed at 200 °C and held for 1 min in a vacuum oven. To protect samples from oxidation, experiments were all carried out under a nitrogen atmosphere. Small amplitude oscillatory shear (SAOS) from 100 to 0.1 rad · s^−1^ was performed in a temperature range from 260 to 140 °C (PE1.1M) and 220 to 140 °C (PE0.5M) with 1% strain. Master curves were achieved by time-temperature superposition (TTS), taking 140 °C as the reference temperature [32].

### 2.5. Solid-State NMR

The measurements were conducted on a Bruker Minispec mq20 low-field spectrometer (Bruker, Billerica, MA, USA) with a proton resonance frequency of 20 MHz. The sample temperature (from 30 °C to 190 °C) was controlled with a BVT3000 heater (Bruker) with a flow of heated nitrogen. The minispec has a typical π/2 pulse length of 3.0 μs and a receiver dead time of 10 μs. The PE samples were loaded into 10 mm diameter NMR tubes and placed in an argon glove box to remove oxygen and sealed completely to prevent oxidation at a high temperature. 

#### 2.5.1. ^1^H MSE and ^1^H Hahn Echo NMR Measurements

MSE was mainly used to retrieve the signals of rigid components lost in single-pulse excitation experiments due to the dead time of the instrument receiver [33,34,35]. Combined with the signal from Hahn echo, we could get the fully refocused FID, which held the information from rigid to mobile parts [36,37].

#### 2.5.2. ^1^H DQ NMR Measurements

DQ signals were excited by Baum-Pines pulses [38,39]. Then, we got double quantum (*I*_DQ_) and reference (*I*_ref_) signals with the growth of DQ evolution time (*τ*_DQ_), from which normalized DQ (*I*_nDQ_) could be obtained, and residual dipole coupling (*D*_res_) (mainly originating from entanglements) could be obtained by fitting *I*_nDQ_ curve. It should be noted that the buildup rate of *I*_nDQ_ is positively correlated with the entanglement density, and *D*_res_ could reflect the structural heterogeneity of the entanglement network [40].

## 3. Results and Discussion

### 3.1. Crystallization

The value of [*η*]c was a typical way to classify intermolecular interpenetration, regardless of its molecular weight [28,29,30]. The degree of entanglement could be partially retained by freeze-extracting [25,26,27]. As listed in Table S1 (see in Supplementary Materials), PE1.1M—0.1 wt% and PE0.5M—0.1 wt% were taken as less-entangled PE, in which intramolecular crystal nucleation happened. While in well-entangled PE, e.g., PE1.1M—5 wt% and PE0.5M—10 wt%, intramolecular crystal nucleation occurred.

**Figure 1:**
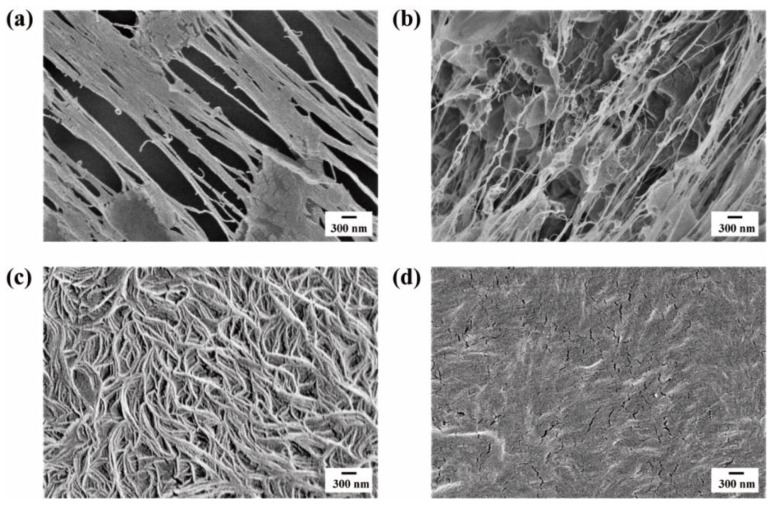
SEM images of (**a**) PE1.1M—0.1 wt%, (**b**) PE0.5M—0.1 wt%, (**c**) PE1.1M—5 wt% and (**d**) PE0.5M—10 wt%.

The crystal morphology of all the PE samples was recorded by SEM, as shown in Figure 1. The fibrils in the less-entangled samples (Figure 1a,b) were directly connected to several crystalline lamellae, which were rarely observed in the well-entangled samples (Figure 1c,d). For less-entangled samples, intramolecular nucleation was kinetically preferred compared with intermolecular crystallization [41,42]. During freeze-extracting, PE chains precipitated under strong shearing, making the specimens stretch partially. As expected, from a microscopic perspective, crystalline lamellae were stretched into fibrils in less-entangled samples. However, it rarely happened in well-entangled samples with complete intermolecular crystallization.

**Figure 2:**
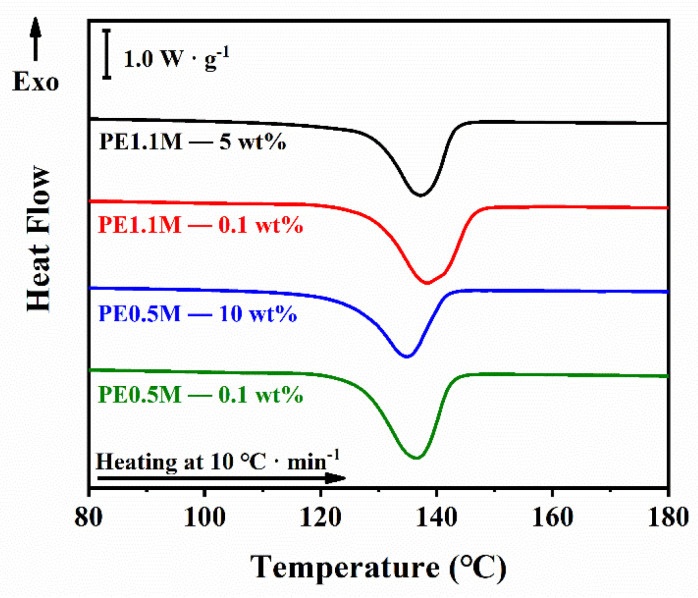
DSC plots four samples with a 10 °C · min^-1^ heating rate in a dry nitrogen atmosphere. For exact values, please refer to Table 1.

DSC tests were performed to further investigate the difference in the crystalline fraction between less-entangled and well-entangled samples, as shown in Figure 2. Melting points (*T*_m_) obtained from DSC are listed in Table 1. Although the crystallization temperatures of all samples were similar, the *T*_m_ of the less-entangled samples were higher than that of the well-entangled samples. According to thermodynamic theory, the *T*_m_ of the crystal increases as the lamellar thickness increases. Therefore, the quantitative relationship between the lamellar thickness and the melting point of the polymer can be described in the Thompson-Gibbs equation [43,44,45]:

(1) $$T_{m}=T_{m}^{0}\left(1-\frac{2\sigma_{e}}{l{∆H}_{0}}\right)$$

where *l*, *T*_m_, *T*_m_^0^, *σ*_e_ and Δ*H*_0_ are the thickness of the crystalline lamella, melting point of the polymer, equilibrium melting point of the polymer, surface free energy, and melting enthalpy of crystal region, respectively. Usually, *T*_m_^0^, *σ*_e_ and Δ*H*_0_ take values of 418.7 K, 90 × 10^−3^ J · m^-2^ and 289 × 10^6^ J · m^-3^ for PE, respectively [31,45].

Lamellar thicknesses (*l*) calculated from Equation (1) are listed in Table 1. All the DSC experiments were repeated three times in our work. As it showed, crystalline lamellae of the less-entangled samples (PE1.1M—0.1 wt% and PE0.5M—0.1 wt%) were thicker than those of the well-entangled samples (PE1.1M—5 wt% and PE0.5M—10 wt%), indicating that molecular chains of less-entangled samples were more easily arranged into thick lamellae without the interference of interpenetration and entanglement from other chains. Meanwhile, the mass crystallinity (*X*_c_) of the less-entangled samples was higher than that of the well-entangled samples, which was consistent with the reduction of the amorphous region where the entanglement was located [22]. The melting peak of PE only decreased very slightly when we changed the heating rate from 1 °C · min^-1^ to 0.1 °C · min^-1^, indicating the similar melting temperature of PE in slow melting to fast melting (10 °C · min^-1^), as shown in Table S2 (see in Supplementary Materials).

According to Flory’s viewpoint [46], for semi-crystalline polymers, the boundary between the ordered crystalline region and the disordered flexible phase was not as clear as that of small molecules. The continuity of the long-chain molecules imposed strong limits on the transition between the perfectly ordered crystalline region and the disordered flexible phase. As a result, a diffuse interfacial region of only a few nanometers in thickness was formed, referred to as the rigid-amorphous intermediate phase here. There were partially ordered chain segments and restricted chain motion compared to the flexible amorphous phase [47]. Therefore, the structure of PE could be divided into a crystalline phase, a rigid-amorphous intermediate phase, and a flexible amorphous phase. Polymer chains in the crystal are arranged closely, and the average proton distance was slightly smaller than that of the mobile amorphous region. Due to the ordered arrangement of the stems in the crystal, the mobility of the chains was greatly restricted, and the protons in the crystal were subject to strong dipolar couplings, which was very suitable for characterizing by FID analysis in low-field solid-state NMR in the following section.

### 3.2. Chain Dynamics

Before we try to analyze chain dynamics affected by entanglement from solid-state NMR, we need to consider the effect of chain relaxation caused by molecular weight. We performed rheological experiments with fully recovered entangled PE samples.

**Figure 3:**
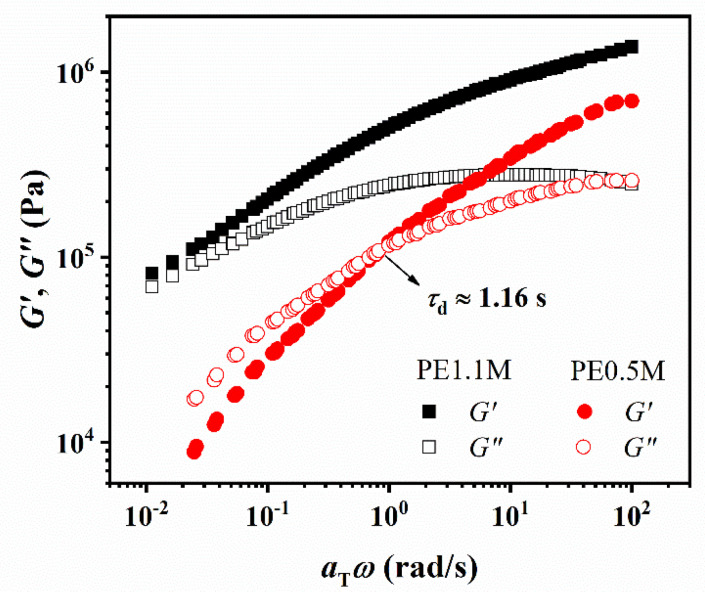
Master curves of PE0.5M and PE1.1M constructed from the SAOS curves at temperatures between 140 and 260 °C at a reference temperature of 140 °C. Solid symbols represent the storage modulus, and hollow symbols represent the loss modulus.

To obtain *τ*_d_ of PE1.1M and PE0.5M, a frequency sweep was performed in the temperature range of 140~260 °C. The master curve with 140 °C as the reference temperature obtained with TTS is shown in Figure 3. Linear viscoelastic relaxation time *τ*_d_ (~1.16 s) for PE0.5M was obtained from the crossover point of storage modulus (G’) and loss modulus (G”) (Figure 3). While for PE1.1M, G’ and G” were gradually approaching in the low-frequency region and the crossover point did not appear. G’ was always larger than G” at the probed temperature range from 140 °C to 260 °C, indicating an elastic behavior. According to the trend, the crossover point was estimated to be smaller than 10^−2^ rad/s, indicating that *τ*_d_ was over 10^2^ s, which was over 2 orders of magnitude larger than the characteristic time for PE0.5M. According to the tube model [48], the larger the molecular weight of the polymer, the more entanglements appear along the chain and the longer the time for a chain to escape from the tube.

**Figure 4:**
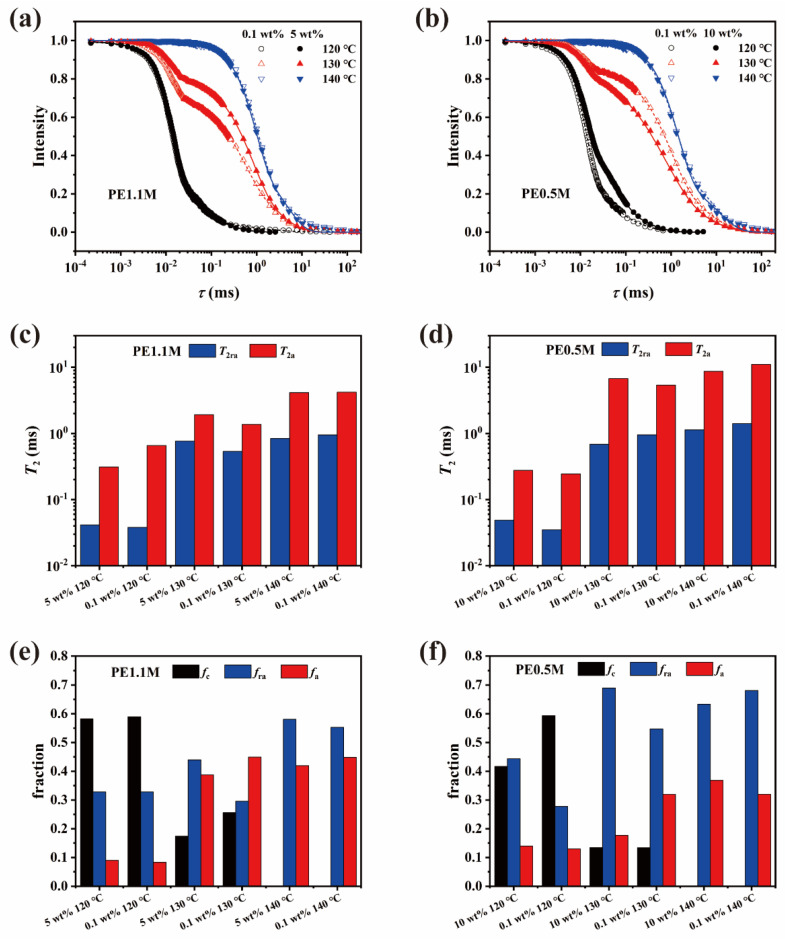
FID signals of (**a**) PE1.1M and (**b**) PE0.5M obtained by MSE and Hahn Echo at temperatures near *T*_m_. The solid and dashed lines represent fitting curves of FID for well-entangled and less-entangled samples, respectively. *T*_2_ of rigid-amorphous intermediate phase (*T*_ra_) and flexible amorphous phase (*T*_a_), a fraction of crystalline phase (*f*_c_), rigid-amorphous intermediate phase (*f*_ra_) and amorphous flexible phase (*f*_a_) obtained by FID fitting with Equation (2) are located at (**c**–**f**).

Besides rheology measurement, fully refocused FID was sensitive in reflecting chain segment mobility in low-field solid-state NMR. As mentioned before, the components of PE could be divided into three phases. The apparent spin-spin relaxation time *T*_2_ of the crystalline phase was short, about 20 μs, implying the absence of chain motions. However, at temperatures well above the glass transition temperature (*T*_g_), the chains in the flexible amorphous phase of the semi-crystalline polymer showed a fast, almost isotropic mobility, leading to a largely averaged dipolar coupling interaction. This averaging led to a slow FID decay and a large apparent spin-spin relaxation *T*_2_. Since the *T*_g_ of about 150 K for PE [49] was well below our measured temperature range, the FID signal could be fitted with Equation (2) to distinguish three components with a large difference in *T*_2_ [23].

(2) $$f\left(t\right)=f_{c}e^{-0.5{\left(at\right)}^{2}}\frac{\sin bt}{bt}+f_{ra}e^{-{\left(\frac{t}{T_{2ra}}\right)}^{v_{ra}}}+f_{a}e^{-{\left(\frac{t}{T_{2a}}\right)}^{v_{a}}}$$

wherein *T*_2ra_, *T*_2a_ denote the apparent spin-spin relaxation time of the rigid-amorphous phase and amorphous phase, respectively; *f*_c_, *f*_ra,_ and *f*_a_ denote the fraction of crystalline phase, rigid-amorphous intermediate phase and flexible amorphous phase, respectively [34]. The fully refocused FID and its fitting results are shown in Figure 4. And the *f*_c,30 °C_ value was listed in Table 1. The *f*_c_ value of the less-entangled PE was higher than those of the well-entangled PE, which agreed well with the trend of the *X*_c_ measured by DSC. Fully refocused FID from 30 °C to 110 °C showed monotonic changes, as shown in Figure S2 (see in Supplementary Materials). We compared FID of all the samples around melting point in the following.

In Figure 4a, as temperature increased, the FID curves shifted to a longer evolution time (*τ*) because of enhanced chain mobility with increased temperature. At 130 °C, the FIDs of the less-entangled and well-entangled samples differed a lot since the melting of the crystals occurred between 120 °C and 140 °C where the melting peak of DSC was located (Figure S1b, see in Supplementary Materials). Crystals gradually melted, then transformed to the rigid-amorphous intermediate phase at 130 °C. With the relaxation of chain segments, chains in the rigid-amorphous phase entered the flexible phase. Therefore, FID curves of PE1.1M—0.1 wt% and PE1.1M—5 wt% differed significantly with a faster decaying rate of the crystalline component in the former. As mentioned before, the crystalline lamellae of PE1.1M—0.1 wt% were thicker than that of PE1.1M—5 wt% (as shown in Table 1), so the former required a higher temperature or longer time to melt. From Figure 4e, a larger proportion of crystalline phase was left in PE1.1M—0.1 wt% at 130 °C. When the temperature rose to 140 °C, both PE1.1M samples completed melting, resulting in two FID curves recovering overlapping.

However, HDPE and UHMWPE exhibited different behaviors around *T*_m_ on fully refocused FID, as shown in Figure 4b. Before melting, the FID of PE0.5M—10 wt% decayed slightly more slowly than the FID of PE0.5M—0.1 wt% because chains attached to thin crystalline lamellae showed faster mobility. Moreover, the FID decay of PE0.5M—10 wt% was faster than that of PE0.5M—0.1 wt% at 130 °C, which was in contradiction to the trend of PE1.1M samples. It was noteworthy that chain mobility around *T*_m_ was related to the rate of chain detachment from the crystal surface and chain relaxation from rigid-amorphous phase to flexible amorphous phase. For PE0.5M samples, the difference in FID decays started from around the evolution time of the rigid-amorphous intermediate phase (0.02~0.1 ms) since it took rigid-amorphous chains of well-entangled HDPE more time than less-entangled one to relax from the constraint of entanglements, which could be seen from the width of DSC peak during heating slowly (Figure S1b, see in Supplementary Materials). However, since the reptation time of UHMWPE was very long (Figure 3), the decisive step in UHMWPE melting depended on the time for chain detachment from the lamellae surface, resulting in different behaviors during melting from HDPE. After melting completely at 140 °C, the FIDs of HDPE overlapped with each other like UHMWPE.

In summary, we captured the intervention of entanglement in the PE melting process. Slow melting was a two-step process: in the first step, the chain detached from the crystalline lamellae surface, and then the detached chain relaxed from the rigid-amorphous phase into the flexible amorphous phase in the second step. On the one hand, for UHMWPE, thicker lamellae in less-entangled samples took longer than well-entangled ones to melt. On the other hand, for HDPE, the time for rigid-amorphous chains of less-entangled samples to relax into a flexible amorphous phase was shorter than the well-entangled one due to the lack of entanglement constraints.

### 3.3. Entanglement Network

^1^H DQ NMR was robust to elucidate residual dipolar coupling, determining entanglement or crosslink densities in linear entangled polymer melts [50]. To further investigate the microscopic mechanism of the PE melting process, we tracked the entanglement network during PE melting with the help of ^1^H DQ NMR. The residual dipolar coupling of PE was influenced by physical entanglements in the amorphous phase and the rigid amorphous phase near the crystalline lamellae, which served as cross-linking points. To reduce the influence of the polymer crystals, ^1^H DQ NMR was performed at temperatures near and above the melting point from 120 °C to 190 °C.

**Figure 5:**
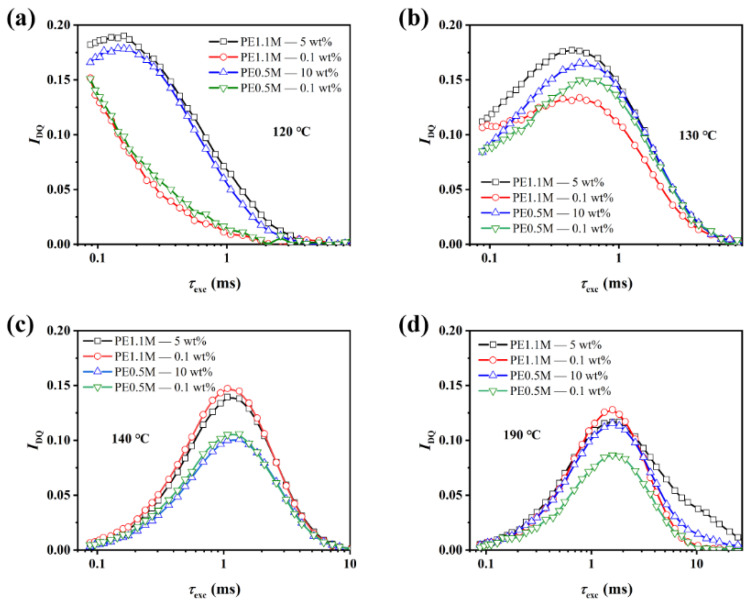
Original DQ buildup curves of all the PE samples at (**a**): 120 °C, (**b**): 130 °C, (**c**): 140 °C and (**d**): 190 °C.

DQ NMR recorded the DQ buildup signals as a function of DQ excitation time, as shown in Figure 5. For less-entangled samples at 120 °C, the mobility of amorphous chains was highly constrained due to the constraint of crystals, leading to the absence of a DQ peak in the original DQ curve because of minimum DQ excitation time was around 100 μs. However, such problems did not exist in well-entangled samples, as shown in Figure 5a. According to DSC, the crystallinity of the less-entangled samples was only about 3% lower than that of well-entangled samples. It was because less-entangled PE was crystallized mainly by intramolecular nucleation, with the amorphous chains separated by loosely distributed crystalline phases and strongly bound by crystalline lamellae. Once crystallites began to melt, the constraint on the chain mobility was released. Figure 5b showed a slight difference in the DQ excitation time of four PE samples corresponding to the peaks of DQ signals at 130 °C. The difference will be more significant after a point-by-point normalization in the following. At 140 °C and above temperature, differences in the DQ excitation time of four samples corresponding to the peaks of DQ signals were not significant with the complete melting of PE, as shown in Figure 5c,d.

Since *I*_DQ_ was subjected to a severe relaxation effect at a long DQ excitation time, *I*_nDQ_ was normalized from the original *I*_DQ_ and *I*_ref_. The initial growth slope of the nDQ curve was positively correlated with the entanglement density. With the nDQ formula of the generalized fitting function (3) [51], we transformed the nDQ buildup curve into a *D*_res_ distribution curve, where the median of the *D*_res_ distribution curve (*D*_med_) indicated the entanglement density and the half-height width *σ* indicated the homogeneity of the entanglements. The larger the *D*_med_, the larger the entanglement density. The smaller the *σ*, the more homogeneous the distribution of entanglements. Detailed procedures were provided in the Supplementary Materials.

(3) $$I_{nDQ}\left(\tau_{DQ}\right)=\int I_{DQ}\left(\tau_{DQ},D_{res}\right)P\left(D_{res}\right)dD_{res}$$

**Figure 6:**
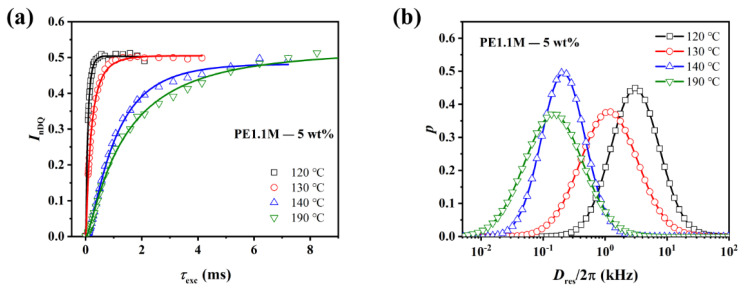
Temperature dependence of (**a**) nDQ curves and (**b**) *D*_res_ distribution curves for PE1.1M—5 wt%. The solid lines in (**a**) represent fitting curves of *I*_nDQ_.

Figure 6a shows nDQ curves normalized from the original DQ and reference intensity for PE 1.1M—5 wt%. From 120 °C to 140 °C, the melting of crystalline lamellae released molecular chains into the amorphous phase and constraints of the crystalline phase on chain relaxation gradually disappeared, leading to nDQ growth slowing down and decrease of *D*_med_ (Figure 6a,b). There was a leap between nDQ curves of 130 °C and 140 °C, where melting has been completed. It was consistent with the temperature range where the peak of the DSC curve disappeared, as shown in Figure S1b (see in Supplementary Materials). However, the nDQ growth rate decreased continuously at 190 °C because of the reptation of chains [52,53]. Other samples showed the same trend, as shown in Figure S3 (see in Supplementary Materials). Heterogeneously distributed chain entanglements have been observed in partially melted PE. As shown in Figure 6b, the *D*_res_ distribution curve widened at 130 °C, for the original disentangled segments and recovering entanglements coexisted in the same polymer melts.

**Figure 7:**
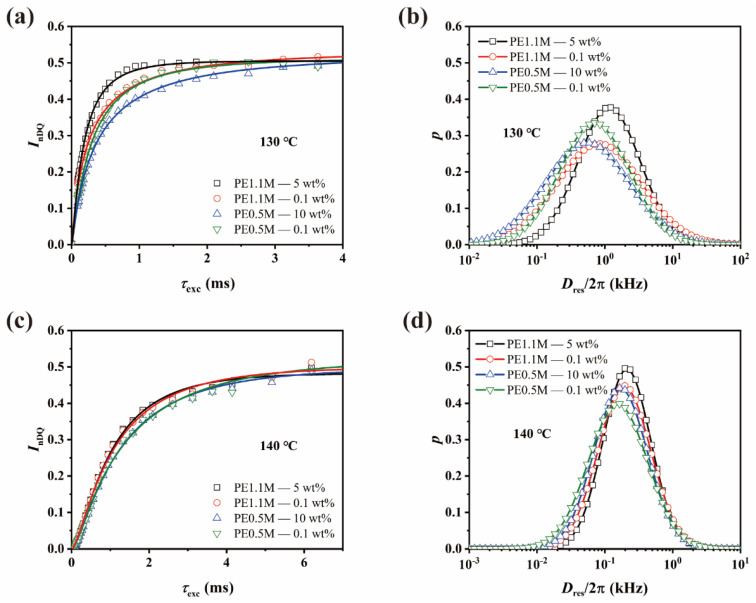
nDQ curves and *D*_res_ distribution of all the PE samples at (**a**,**b**): 130 °C and (**c**,**d**): 140 °C. The solid lines in (**a**) and (**c**) represent fitting curves of *I*_nDQ_. For 120 °C and 190 °C, please refer to Figure S5 (see in Supplementary Materials).

To investigate the entanglement network evolution during melting, we compared the nDQ and *D*_res_ distribution curves of four samples at 130 °C and 140 °C (Figure 7). 

All the samples were under slow melting at 130 °C. As discussed previously, it took rigid-amorphous chains of PE1.1M samples a lot more time to relax and re-entangle than PE0.5M samples after they detached from the crystalline lamellae because the chain reptation time of PE1.1M was much longer than that of PE0.5M. So, the growth rate of nDQ curves of PE1.1M could reflect the original degree of entanglement. However, when the molecular chain of PE0.5M—0.1 wt% detached from the lamella, it started to creep and entangle with other chains. The original degree of entanglement retained from freeze-extracting could not be obtained from the growth rate of nDQ curves. Therefore, we got a larger *D*_med_ in PE0.5M—0.1 wt% than PE0.5M—10 wt%, as shown in Figure 7b.

At 140 °C, crystalline lamellae have just melted, resulting in the absence of permanent confinement of chain mobility. The residual dipolar coupling was contributed only by inter-segment proximity, including topological entanglements. The chain segments moved faster and could free themselves from physical topological constraints as the temperature rose [50]. This meant a decrease in network-like protons was expected, as shown in Figure S4 (see in Supplementary Materials). The original degree of entanglement still contributed to residual dipolar coupling in PE1.1M samples. However, the difference between less-entangled and well-entangled ones was narrowing, for the entanglement networks were diluted with increasing isotropic moieties. Polymers packed with each other in melts no matter how many entanglements are involved in the melt state, and every inter-segment proximity accounted for the residual dipolar coupling. In contrast, *D*_res_ distribution curves of PE0.5M samples almost overlapped since homogeneous melts had been formed after reptation (Figure 7d), regardless of initial entanglement states.

When the temperature rose to 190 °C, the motion of molecular chains was more drastic. From Figure S4 (see in Supplementary Materials), it could be seen that the number of network-like protons of PE1.1M samples continued to decrease, which was consistent with the trend at 140 °C. While the number of network-like protons of PE0.5M samples began to level off due to the recovery of entanglements caused by the fast reptation of molecular chains. It agreed well with what was observed in partially cross-linked HDPE [50]. 

These results corresponded to our previous conjecture on the melting process of PE. For PE with different lengths of molecular chains, entanglements are involved in the melting process by different mechanisms.

**Figure 8:**
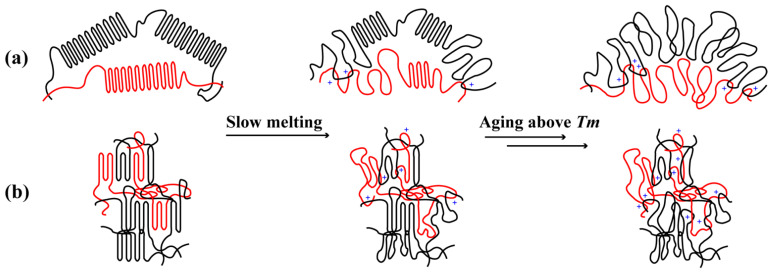
Schematic transformation of PE morphologies: (**a**) less-entangled UHMWPE and (**b**) well-entangled UHMWPE during slow melting. The left column shows the initial morphology of PE from freeze-extracting. The middle column shows the state of PE in slow melting, and the right column shows their melting states completely. The red line represents the observed chain, the black line represents its neighbors, and the blue cross represents the new entanglement during slow melting.

Figure 8 illustrates the evolution of entanglements during the slow melting of UHMWPE according to previous results. In the beginning, crystals of PE were prepared from dilute solutions predominantly composed of a single isolated chain, regarded as intramolecular nucleated [21,23]. While crystals of well-entangled samples comprised different polymer chains, regarded as intermolecular nucleated. The number of entanglements along the chain of well-entangled samples was larger than that of less-entangled ones.

The amorphous state of chains includes rigid amorphous and flexible amorphous chains. When UHMWPE melted slowly, the chain segmental mobility in the amorphous state of less-entangled UHMWPE was slower than that of well-entangled one according to *T*_2ra_ in Figure 4c, mainly resulting from more residual crystalline lamellae (see *f*_c_ in Figure 4c), as Figure 8 schematically illustrated. However, when UHMWPE was aging above the melting point, their chain mobility was generally accelerated. As a result, chain segmental relaxation and re-entanglement co-occurred. In a similar nonequilibrium disentangled polystyrene samples, rheological experiments revealed that the re-entanglement time is 2–4 orders of magnitude greater than the reptation time, which was considered an extension of the Doi-Edwards relaxation model [54]. In de Gennes’s work [55], he supposed a kind of tight knot with an unexpectedly long life, which also increased difficulty in processing, yet differed a lot from topological entanglements in our solution-crystallized PE. Rastogi et al. found the time required for a disentangled PE with *M*_w_ of 1400 kg/mol to reach 98% of its maximum plateau modulus was around 15,000 s [12], which was approximately three orders of magnitude larger than the *τ*_d_ of PE1.1M we obtained in the rheological experiment. Due to the extra-long reptation time, UHMWPE’s initial degree of entanglement was maintained partially while HDPE couldn’t.

The initial crystallinity information can be well retained during conventional fast melting, e.g., 10 °C · min^−1^. However, an immediate increase in entropy allows homogeneous polymer melts to form quickly, and entanglement has little effect on the melting mechanism of the polymer [13]. The evolution of entanglement can only be detected during slow melting. Notably, the time required to erase the entanglement memory varies with the molecular weight of PE.

## 4. Conclusions

Herein this work, we got PE with different degrees of entanglement by freeze-extracting. With less entanglement preferred intramolecular-nucleated crystallization, PE showed a higher crystallinity and thicker crystalline lamellae before melting. With the help of fully refocused ^1^H FID in low-field solid-state NMR, we found entanglement restricted the chain segments’ relaxation in an amorphous state and then determined their melting behavior. The longer the PE chain is in a less-entangled state, the harder the process of merging into mobile parts after detaching from crystalline lamella during melting. ^1^H DQ NMR was further used to obtain information caused by residual dipolar interaction. A significant difference in the DQ signals of less-entangled and well-entangled PE was captured before melting, independent of molecular weight. It is because the entanglement network in less-entangled PE was greatly disturbed by intramolecular-nucleated crystals. During melting, less-entangled UHMWPE could keep disentangled while less-entangled HDPE couldn’t. Unfortunately, no noticeable difference was found in DQ experiments between PE melts with different degrees of entanglement after melting. This was ascribed to the small contribution of entanglements compared with total inter-segment proximity in melts. Overall, less-entangled UHMWPE could reserve its disentangled state around the melting point long enough to achieve a better way of processing.

## Figures and Tables

**Table 1:** Melting point (*T*_m_), mass crystallinity (*X*_c_) and lamellar thickness (*l*) of all the samples obtained from DSC. The fraction of the crystalline phase at 30 °C (*f*_c, 30 °C_) was fitted from the low-field NMR.

Samples	*f*_c, 30 °C_ (%)	*T*_m_ (°C)	*X*_c_ (%)	*l*/nm
PE1.1M—0.1 wt%	70.7 ± 1.7	139.2 ± 0.9	38.1 ± 3.3	41.9 ± 6.5
PE1.1M—5 wt%	64.2 ± 1.7	137.8 ± 0.6	34.8 ± 3.1	34.0 ± 2.7
PE0.5M—0.1 wt%	70.0 ± 1.9	136.5 ± 0.2	37.8 ± 1.4	28.9 ± 0.7
PE0.5M—10 wt%	59.2 ± 0.6	135.8 ± 0.6	34.5 ± 2.2	27.0 ± 1.7

## Data Availability

The data presented in this study are available upon request from the corresponding author.

## Supplementary Materials

The following supporting information can be downloaded at: https://www.mdpi.com/article/10.3390/polym15081910/s1, Figure S1: DSC plots of four samples with a heating rate of (a): 1 °C·min^-1^ and (b): 0.1 °C·min^-1^ in a dry nitrogen atmosphere; Figure S2: Fully refocused FID signals of (a): PE1.1M—5 wt%, (b): PE1.1M—0.1 wt%, (c): PE0.5M—10 wt% and (d): PE0.5M—0.1 wt%; Figure S3: Dependence of nDQ and Dres curves of (a,b): PE1.1M—0.1 wt%, (c,d): PE0.5M—10 wt%, (e,f): PE0.5M—0.1 wt% with temperature. The solid lines in (a), (c) and (e) represent fitting curves of *I*_nDQ_; Figure S4: Fraction of the network-like protons as a function of temperature obtained by fitting based on Equation S3; Figure S5: Comparison of nDQ buildup curves from all of the samples at (a,b): 120 °C and (c,d): 190 °C. The solid lines in (a) and (c) represent fitting curves of *I*_nDQ_; Table S1: PE used in experiments and their corresponding characteristics, including weight-average molar mass (*M*_w_), polydispersity (PDI), viscosity-average molar mass (*M*_η_) and [*η*]c; Table S2: The melting point (*T*_m_) and the crystallinity (*X*_c_) obtained by DSC plots. References [56,57,58,59,60] are cited in the Supplementary Materials.

## References

[1] Kurtz S.M. (2015). Editorial Comment: Advances in UHMWPE Biomaterials. Clin. Orthop. Relat. Res..

[2] Kurtz S.M. (1999). Advances in the processing, sterilization, and crosslinking of ultra-high molecular weight polyethylene for total joint arthroplasty. Biomaterials.

[3] Li S., Burstein A.H. (1994). Ultra-high molecular weight polyethylene. The material and its use in total joint implants. J. Bone Jt. Surg..

[4] Gao Q., Hu J.-T., Yang Y., Wang M., Zhang M., Tang Z., Zhang M., Liu W., Wu G.-Z. (2018). Fabrication of New High-Performance UHMWPE-Based Conductive Fibers in a Universal Process. Ind. Eng. Chem. Res..

[5] Liu S., Zhou C., Yu W. (2011). Phase separation and structure control in ultra-high molecular weight polyethylene microporous membrane. J. Membr. Sci..

[6] Oral E., Wannomae K.K., Hawkins N., Harris W.H., Muratoglu O.K. (2004). α-Tocopherol-doped irradiated UHMWPE for high fatigue resistance and low wear. Biomaterials.

[7] Liu X., Yu W. (2020). Role of chain dynamics in the melt memory effect of crystallization. Macromolecules.

[8] Milner S.T., McLeish T. (1998). Reptation and Contour-Length Fluctuations in Melts of Linear Polymers. Phys. Rev. Lett..

[9] Psarski M., Piorkowska E., Galeski A. (2000). Crystallization of Polyethylene from Melt with Lowered Chain Entanglements. Macromolecules.

[10] Rastogi S., Spoelstra A.B., Goossens J.G.P., Lemstra P.J. (1997). Chain Mobility in Polymer Systems: On the Borderline between Solid and Melt. 1. Lamellar Doubling during Annealing of Polyethylene. Macromolecules.

[11] Everaers R., Sukumaran S.K., Grest G.S., Svaneborg C., Sivasubramanian A., Kremer K. (2004). Rheology and Microscopic Topology of Entangled Polymeric Liquids. Science.

[12] Pandey A., Champouret Y., Rastogi S. (2011). Heterogeneity in the Distribution of Entanglement Density during Polymerization in Disentangled Ultrahigh Molecular Weight Polyethylene. Macromolecules.

[13] Rastogi S., Lippits D.R., Peters G.W.M., Graf R., Yao Y., Spiess H. (2005). Heterogeneity in polymer melts from melting of polymer crystals. Nat. Mater..

[14] Wool R.P. (1993). Polymer entanglements. Macromolecules.

[15] Pawlak A. (2019). The Entanglements of Macromolecules and Their Influence on the Properties of Polymers. Macromol. Chem. Phys..

[16] Kong D., Yang M., Zhang X., Du Z., Fu Q., Gao X., Gong J. (2021). Control of Polymer Properties by Entanglement: A Review. Macromol. Mater. Eng..

[17] Pawlak A., Krajenta J., Galeski A. (2017). The crystallization of polypropylene with reduced density of entanglements. J. Polym. Sci. Part B Polym. Phys..

[18] Schulz M., Schäfer M., Saalwächter K., Thurn-Albrecht T. (2022). Competition between crystal growth and intracrystalline chain diffusion determines the lamellar thickness in semicrystalline polymers. Nat. Commun..

[19] Li Z., Hong Y.-L., Yuan S., Kang J., Kamimura A., Otsubo A., Miyoshi T. (2015). Determination of Chain-Folding Structure of Isotactic Polypropylene in Melt-Grown α Crystals by 13C–13C Double Quantum NMR and Selective Isotopic Labeling. Macromolecules.

[20] Schulz M., Seidlitz A., Kurz R., Bärenwald R., Petzold A., Saalwächter K., Thurn-Albrecht T. (2018). The Underestimated Effect of Intracrystalline Chain Dynamics on the Morphology and Stability of Semicrystalline Polymers. Macromolecules.

[21] Yao Y.-F., Graf R., Spiess H., Rastogi S. (2008). Restricted Segmental Mobility Can Facilitate Medium-Range Chain Diffusion: A NMR Study of Morphological Influence on Chain Dynamics of Polyethylene. Macromolecules.

[22] Rastogi S., Yao Y., Ronca S., Bos J., van der Eem J. (2011). Unprecedented High-Modulus High-Strength Tapes and Films of Ultrahigh Molecular Weight Polyethylene via Solvent-Free Route. Macromolecules.

[23] Bärenwald R., Champouret Y., Saalwächter K., Schäler K. (2012). Determination of Chain Flip Rates in Poly(ethylene) Crystallites by Solid-State Low-Field 1H NMR for Two Different Sample Morphologies. J. Phys. Chem. B.

[24] Litvinov V., Deblieck R., Clair C., Fonteyne W.V.D., Lallam A., Kleppinger R., Ivanov D.A., Ries M.E., Boerakker M. (2020). Molecular Structure, Phase Composition, Melting Behavior, and Chain Entanglements in the Amorphous Phase of High-Density Polyethylenes. Macromolecules.

[25] Sun Q., Fu Q., Xue G., Chen W. (2001). Crystallization Behavior of Syndiotactic Poly(propylene) Freeze-Dried from Toluene at Very Dilute Concentration. Macromol. Rapid Commun..

[26] Chen J., Sun Q., Zou Y., Xue G. (2002). DSC studies on the melting crystallization of polyethylenes prepared from alkanes of varying molecular size. Polymer.

[27] Xiao Z., Sun Q., Xue G., Yuan Z., Dai Q., Hu Y. (2003). Thermal behavior of isotactic polypropylene freeze-extracted from solutions with varying concentrations. Eur. Polym. J..

[28] Frisch H.L., Simha R., Eirich F.R. (1956). Chapter 14—The Viscosity of Colloidal Suspensions and Macromolecular Solutions. Rheology.

[29] Wang X., Tao F., Sun P., Zhou D., Wang Z., Gu Q., Hu J., Xue G. (2007). Probing Chain Interpenetration in Polymer Glasses by 1H Dipolar Filter Solid-State NMR under Fast Magic Angle Spinning. Macromolecules.

[30] Chang L.P., Morawetz H. (1987). Study of the interpenetration of monodisperse polystyrene in semidilute solution by fluorescence after freeze-drying. Macromolecules.

[31] Li W., Yue Z., Lozovoi A., Petrov O., Mattea C., Stapf S. (2018). Heterogeneous distribution of chain mobility in nascent UHMWPE in the less entangled state. J. Polym. Res..

[32] Ahmadi M., Seiffert S. (2021). Coordination Geometry Preference Regulates the Structure and Dynamics of Metallo-Supramolecular Polymer Networks. Macromolecules.

[33] Maus A., Hertlein C., Saalwächter K. (2006). A Robust Proton NMR Method to Investigate Hard/Soft Ratios, Crystallinity, and Component Mobility in Polymers. Macromol. Chem. Phys..

[34] Mauri M., Thomann Y., Schneider H., Saalwächter K. (2008). Spin-diffusion NMR at low field for the study of multiphase solids. Solid State Nucl. Magn. Reson..

[35] Schäler K., Ostas E., Schröter K., Thurn-Albrecht T., Binder W.H., Saalwächter K. (2011). Influence of Chain Topology on Polymer Dynamics and Crystallization. Investigation of Linear and Cyclic Poly(ε-caprolactone)s by 1H Solid-State NMR Methods. Macromolecules.

[36] Litvinov V.M., Orza R.A., Klüppel M., van Duin M., Magusin P.C.M.M. (2011). Rubber–Filler Interactions and Network Structure in Relation to Stress–Strain Behavior of Vulcanized, Carbon Black Filled EPDM. Macromolecules.

[37] Gao Y., Zhang R., Lv W., Liu Q., Wang X., Sun P., Winter H.H., Xue G. (2014). Critical Effect of Segmental Dynamics in Polybutadiene/Clay Nanocomposites Characterized by Solid State 1H NMR Spectroscopy. J. Phys. Chem. C.

[38] Lange F., Schwenke K., Kurakazu M., Akagi Y., Chung U.-I., Lang M., Sommer J.-U., Sakai T., Saalwächter K. (2011). Connectivity and Structural Defects in Model Hydrogels: A Combined Proton NMR and Monte Carlo Simulation Study. Macromolecules.

[39] Chassé W., Lang M., Sommer J.-U., Saalwächter K. (2011). Cross-Link Density Estimation of PDMS Networks with Precise Consideration of Networks Defects. Macromolecules.

[40] Wang H., Peng W.-S., Wu Q., Zhao Y., Wang S.-T., Yang Y., Wu J.-R., Wang X.-L., Zhang R.-C. (2022). Interplay of Crosslinking Structures and Segmental Dynamics in Solid-Liquid Elastomers. Chin. J. Polym. Sci..

[41] Hoffman J.D., Miller R.L. (1997). Kinetic of crystallization from the melt and chain folding in polyethylene fractions revisited: Theory and experiment. Polymer.

[42] Hu W., Reiter G., Strobl G.R. (2007). Intramolecular Crystal Nucleation. Progress in Understanding of Polymer Crystallization.

[43] Hoffman J.D., Lauritzen J.I. (1961). Crystallization of bulk polymers with chain folding: Theory of growth of lamellar spherulites. J. Res. Natl. Bur. Stand. Sect. A Phys. Chem..

[44] Hoffman J.D., Davis G.T., Lauritzen J.I., Hannay N.B. (1976). The Rate of Crystallization of Linear Polymers with Chain Folding. Treatise on Solid State Chemistry.

[45] Li Z., Ye C., Feng L., Xia J., Zhang L., Zhao W., Hu Y. (2019). Crystal morphology and corresponding physical properties of nascent ultra-high molecular weight polyethylene powder with short-branched chains. Polymer.

[46] Flory P.J. (1962). On the Morphology of the Crystalline State in Polymers. J. Am. Chem. Soc..

[47] Mandelkern L., Alamo R.G., Kennedy M.A. (1990). The interphase thickness of linear polyethylene. Macromolecules.

[48] Doi M., Edwards S.F. (1978). Dynamics of concentrated polymer systems. Part 3.—The constitutive equation. J. Chem. Soc. Faraday Trans. 2.

[49] Röthemeyer F., Sommer F. (2013). Kautschuktechnologie. Kautschuk Technologie: Werkstoffe–Verarbeitung–Produkte.

[50] Shahsavan F., Beiner M., Saalwächter K. (2021). Chain dynamics in partially cross-linked polyethylene by combined rheology and NMR -based molecular rheology. J. Polym. Sci..

[51] Zhang R., Yu S., Chen S., Wu Q., Chen T., Sun P., Li B., Ding D. (2014). Reversible Cross-Linking, Microdomain Structure, and Heterogeneous Dynamics in Thermally Reversible Cross-Linked Polyurethane as Revealed by Solid-State NMR. J. Phys. Chem. B.

[52] Chávez F.V., Saalwächter K. (2011). Time-Domain NMR Observation of Entangled Polymer Dynamics: Universal Behavior of Flexible Homopolymers and Applicability of the Tube Model. Macromolecules.

[53] Chávez F.V., Saalwächter K. (2011). Time-Domain NMR Observation of Entangled Polymer Dynamics: Analytical Theory of Signal Functions. Macromolecules.

[54] Teng C., Gao Y., Wang X., Jiang W., Zhang C., Wang R., Zhou D., Xue G. (2012). Reentanglement Kinetics of Freeze-Dried Polymers above the Glass Transition Temperature. Macromolecules.

[55] De Gennes P.G. (1984). Tight knots. Macromolecules.

[56] Wang H., Yan Z., Li D., Wang X., Zhang R., Herman F.M. (2022). Solid-State NMR Spectroscopy. Encyclopedia of Polymer Science and Technology.

[57] Saalwächter K. (2007). Proton multiple-quantum NMR for the study of chain dynamics and structural constraints in polymeric soft materials. Prog. Nucl. Magn. Reson. Spectrosc..

[58] Lorthioir C., Randriamahefa S., Deloche B. (2013). Some aspects of the orientational order distribution of flexible chains in a diblock mesophase. J. Chem. Phys..

[59] Jakisch L., Garaleh M., Schäfer M., Mordvinkin A., Saalwächter K., Böhme F. (2017). Synthesis and Structural NMR Characterization of Novel PPG/PCL Conetworks Based upon Heterocomplementary Coupling Reactions. Macromol. Chem. Phys..

[60] Zhang R., Zhang C., Yang Z., Wu Q., Sun P., Wang X. (2020). Hierarchical Dynamics in a Transient Polymer Network Cross-Linked by Orthogonal Dynamic Bonds. Macromolecules.

