# Supplementary material for: Chain Dynamics of Partially Disentangled UHMWPE around Melting Point Characterized by 1H Low-Field Solid-State NMR
Source: Polymers (Basel). 2023 Apr 16;15(8):1910. doi: 10.3390/polym15081910 (PMC10142606; doi:10.3390/polym15081910)
Supplement: Supplementary file 1 [file polymers-15-01910-s001.zip › polymers-2323720-SI.pdf]

Supplementary Materials: Chain Dynamics of Partially Disentangled UHMWPE around Melting Point Characterized by ^{1}H Low-Field Solid-State NMR

1. Information about PE freeze-extracted from dilute solution

To make sure no degradation happened, we cross-checked the molecular weight by GPC and Ubbelohde viscometer. Take PE1.1M—0.1 wt% as an example. The value of M_w changed by 1.2% before and after thermal treatment at 135 °C. The relative deviation for M_η of PE1.1M was 2.6%. PE with $[\eta]c$ less than 1 was regarded as less-entangled, while $[\eta]c$ larger than 10 was regarded as well-entangled.

Table S1. PE used in experiments and their corresponding characteristics, including weight-average molar mass (M_w), polydispersity (PDI), viscosity-average molar mass (M_η) and $[\eta]c$.

Samples	M_w (kg/mol)	PDI	M_η (kg/mol)	$[\eta]c$
PE1.1M—0.1 wt%	1088	9.30	1160	0.84
PE1.1M—5 wt%	1149	10.30	1130	41.98
PE0.5M—0.1 wt%	463	18.05	500	0.45
PE0.5M—10 wt%	495	14.84	530	50.43

2. Effect of heating rates on PE melting

All of the NMR experiments at elevated temperatures were at isothermal conditions. So we conducted the DSC experiments with different heating rates. The melting peak of PE only decreased very slightly when we changed the heating rate from 1 °C · min^{-1} to 0.1 °C · min^{-1}.

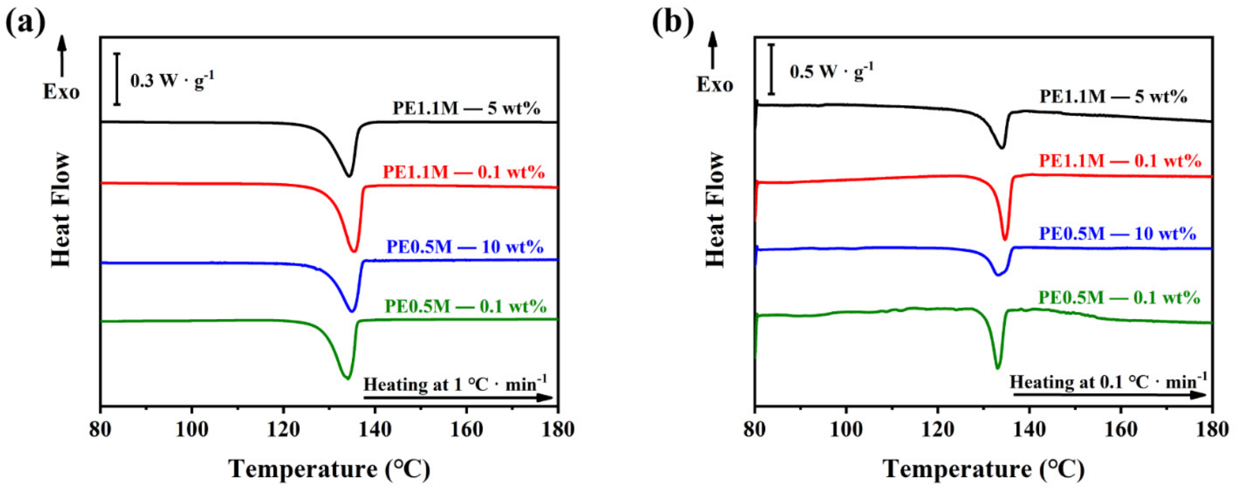

Figure S1. DSC plots four samples with a heating rate of (a): 1 °C · min^{-1} and (b): 0.1 °C · min^{-1} in a dry nitrogen atmosphere.

These results suggested no PE crystal melted before 120 °C, and PE crystal melted totally above 140 °C in corresponding NMR experiments. The melting point (T_m) and the crystallinity (X_c) obtained by integration over the DSC curves are listed in Table S2.

Table S2. The melting point (T_m) and the crystallinity (X_c) obtained by DSC plots.

Samples	$T_{m,\ 1\ °C/min}$ (°C)	$X_{c,\ 1\ °C/min}$ (%)	$T_{m,\ 0.1\ °C/min}$ (°C)	$X_{c,\ 0.1\ °C/min}$ (%)
PE1.1M—0.1 wt%	135.6	42.3	134.7	42.1
PE1.1M—5 wt%	134.3	37.4	133.8	34.1
PE0.5M—0.1 wt%	134.1	38.2	132.9	35.0
PE0.5M—10 wt%	135.0	36.2	133.2	30.1

3. Evolution of fully refocused FID signals from 30 °C to 110 °C

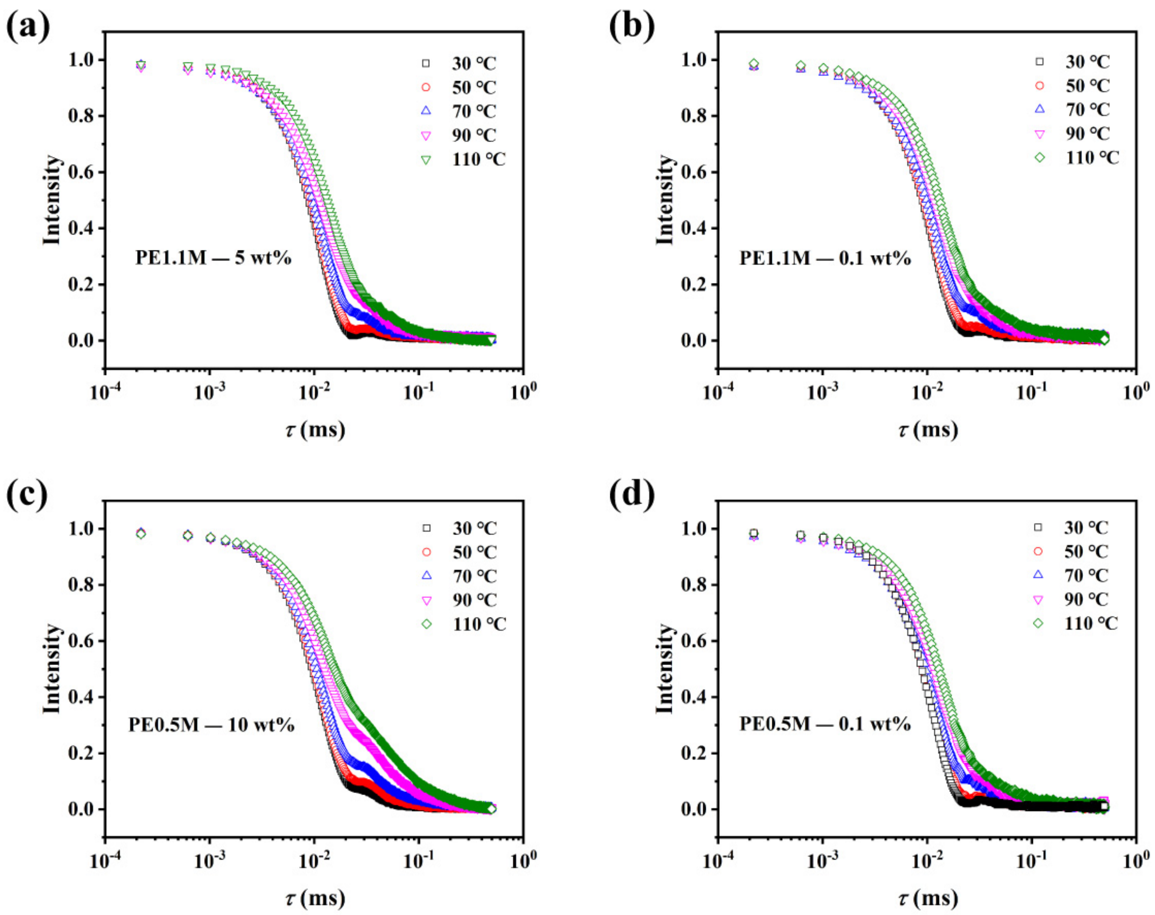

Figure S2. Fully refocused FID signals of (**a**): PE1.1M—5 wt%, (**b**): PE1.1M—0.1 wt%, (**c**): PE0.5M—10 wt% and (d): PE0.5M—0.1 wt%.

Take PE1.1M—5 wt% as an example. As the temperature rose from 30 °C to 110 °C, the intensity of the FID signal decayed more slowly. On the other hand, the signal longer than 0.1 ms ascribed to the behavior of protons in the amorphous regions gradually increased with increasing temperatures [23], as shown in Figure S2. The same trend was found for other PE samples.

4.1. H DQ NMR data analysis

Typically, from the DQ NMR experiments, two datasets could be obtained, I_{DQ} and I_{ref}, corresponding to the DQ and reference signal intensity, respectively [56]. The sum of these two signals forms the sum multiple-quantum (MQ) signal intensity,

$$I_{\sum MQ} = I_{ref} + I_{DQ} \qquad (S1)$$

whose decay as a function of DQ evolution/excitation time (τ_{DQ}) is dominated by segmental fluctuations. Since the DQ intensity is subjected to a severe relaxation effect at a long DQ evolution time, a point-by-point normalization protocol is required to obtain a normalized DQ (nDQ) intensity buildup curve,

$$I_{nDQ}(\tau_{DQ}) = \frac{I_{DQ}(\tau_{DQ})}{I_{\sum MQ}(\tau_{DQ})} \qquad \text{(S2)}$$

Such a normalization procedure could eliminate the effect of temperature-dependent segmental dynamics (which generally induces significant changes in T_1 or T_2 relaxation). Thus the normalized DQ intensity buildup exclusively depends on D_{res} characterizing restrained structures of entanglement networks [57]. However, before the normalization, the signals of isotropic mobile components that did not contribute to the MQ coherent signals, such as dangling/sol chains and loops, had to be subtracted from the sum MQ intensity,

$$I_{\sum MQ} = I_{DQ} + I_{ref} - f_B e^{-2\tau_{DQ}/T_{2B}} - f_c e^{-2\tau_{DQ}/T_{2C}} \qquad \text{(S3)}$$

f_B and f_C are the fractions of two potential separable isotropic mobile components with an apparent spin-spin relaxation time of T_{2B} and T_{2C}, respectively.

To obtain quantitative information, numeric integration needs to be implemented to fit the normalized DQ (nDQ) buildup curves as a function of DQ excitation/evolution time (τ_{DQ}). In most cases, heterogeneity is present in the system, either structural/conformational differences among polymer chains or dynamics gradient/variation in different components. Herein, log-normal distribution [58,59] is often adapted due to its capability to characterize the D_{res} distribution width (σ) as well as the averaged D_{res} values in terms of the median value (D_m). The probability function for the log-normal distribution is,

$$P(D_{res}) = \frac{1}{\sqrt{2\pi}\sigma D_{res}} e^{-\frac{(\ln(D_{res}/D_m))^2}{2\sigma^2}} \qquad \text{(S4)}$$

Here, σ is the standard deviation and dimensionless, reflecting the inhomogeneity of the distribution and roughly corresponding to the FWHM (full width at half maximum) of the D_{res} distribution on a logarithmic scale (Equation S4). D_m is the median value for the D_{res} distribution, indicating the residual dipolar couplings with the largest probability. The finalized nDQ intensity buildup can thus be calculated by a finite-step integral according to [60]

$$I_{nDQ}(\tau_{DQ}) = \int P(D_{res})\, I_{DQ}(\tau_{DQ}, D_{res}) dD_{res} = \int \frac{1}{\sqrt{2\pi}\sigma D_{res}} e^{-\frac{(ln(D_{res}/D_m))^2}{2\sigma^2}} I_{DQ}(\tau_{DQ}, D_{res}) dD_{res}$$

$$= \int \frac{1}{\sqrt{2\pi}\sigma} e^{-\frac{(ln(D_{res}/D_m))^2}{2\sigma^2}} I_{DQ}(\tau_{DQ}, D_{res}) d\ln D_{res} = \int P(\ln D_{res})\, I_{DQ}(\tau_{DQ}, D_{res}) d\ln D_{res} \qquad \text{(S5)}$$

where

$$P(\ln D_{res}) = \frac{1}{\sqrt{2\pi}\sigma} e^{-\frac{(ln(D_{res}/D_m))^2}{2\sigma^2}} \qquad \text{(S6)}$$

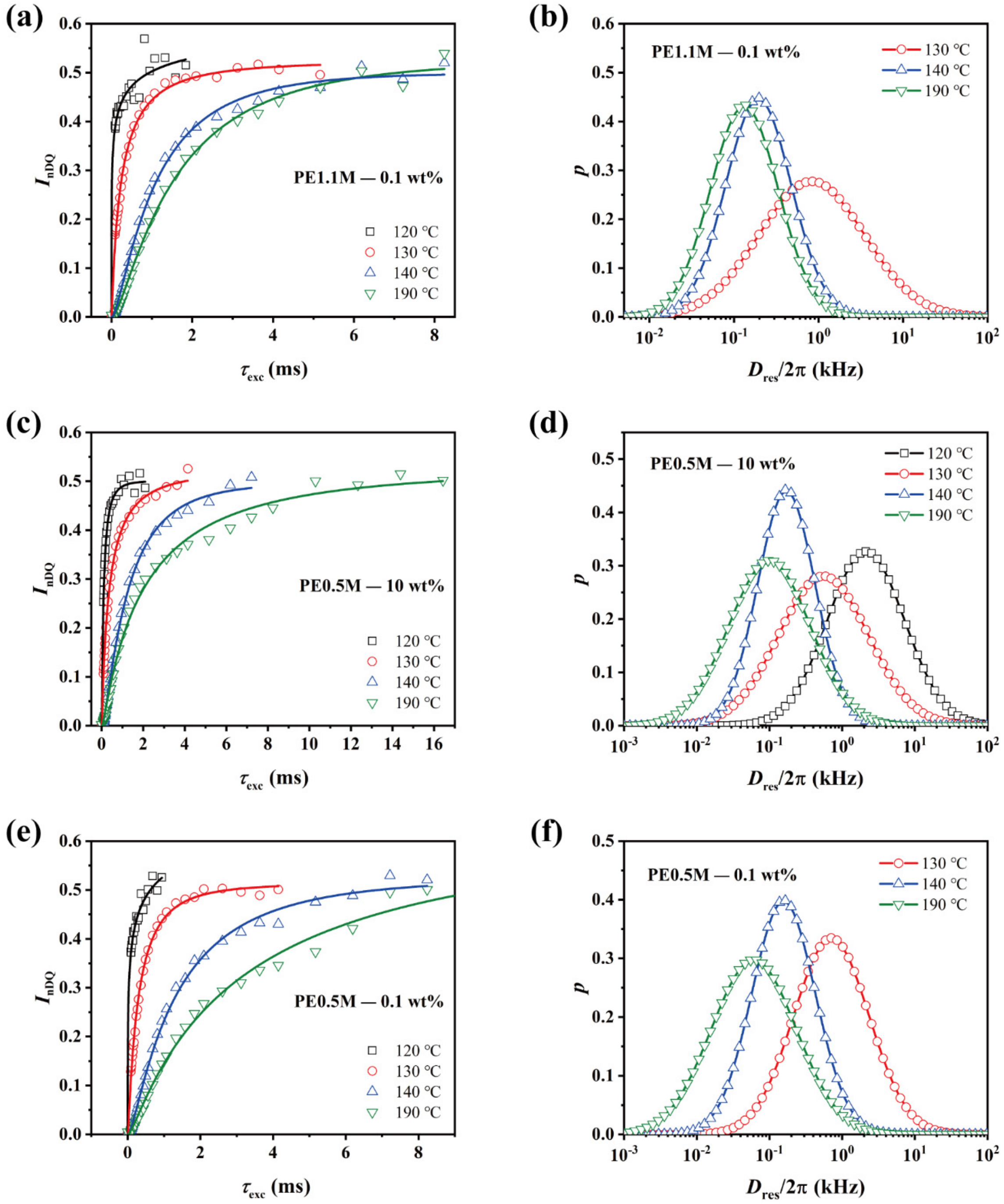

Figure S3. Dependence of nDQ and D_{res} curves of (**a**) (**b**): PE1.1M—0.1 wt%, (**c**) (**d**): PE0.5M—10 wt%, (**e**) (**f**): PE0.5M—0.1 wt% with temperature. The solid lines in (**a**), (**c**) and (**e**) represent fitting curves of I_{nDQ}.

Temperature dependence of the nDQ curves for PE1.1M—0.1 wt%, PE0.5M—10 wt% and PE0.5M—0.1 wt% followed a similar pattern with that for PE1.1M—5 wt%. From 120 °C to 140 °C, nDQ growth slowed down as the melting of crystalline lamellae released molecular chains into the amorphous phase. D_{res} distribution of disentangled samples at 120 °C could not be obtained by fitting nDQ curves because of the absence of a DQ peak in the original DQ curves.

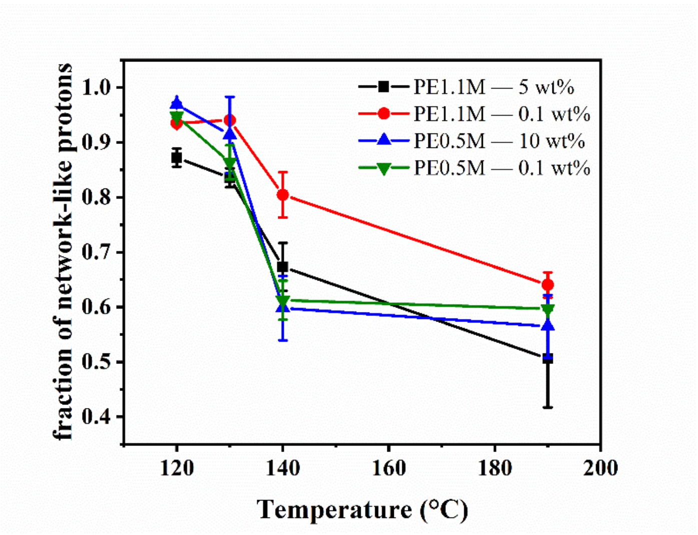

Figure S4. Fraction of the network-like protons as a function of temperature obtained by fitting based on Equation S3.

The fraction of network-like protons reflected the number of coupled protons corresponding to anisotropic segmental motion. By increasing the temperature, the segments moved faster. As a result, they could free themselves from the constraints, which meant that a decrease of network-like protons with a temperature above T_m was expected, similar to what was observed for PE1.1M samples in Figure S4. Meanwhile, chains started to re-entangle with each other once they were released from the old restrictions. As Figure S4 shows, curves of PE0.5M samples began to level off above T_m.

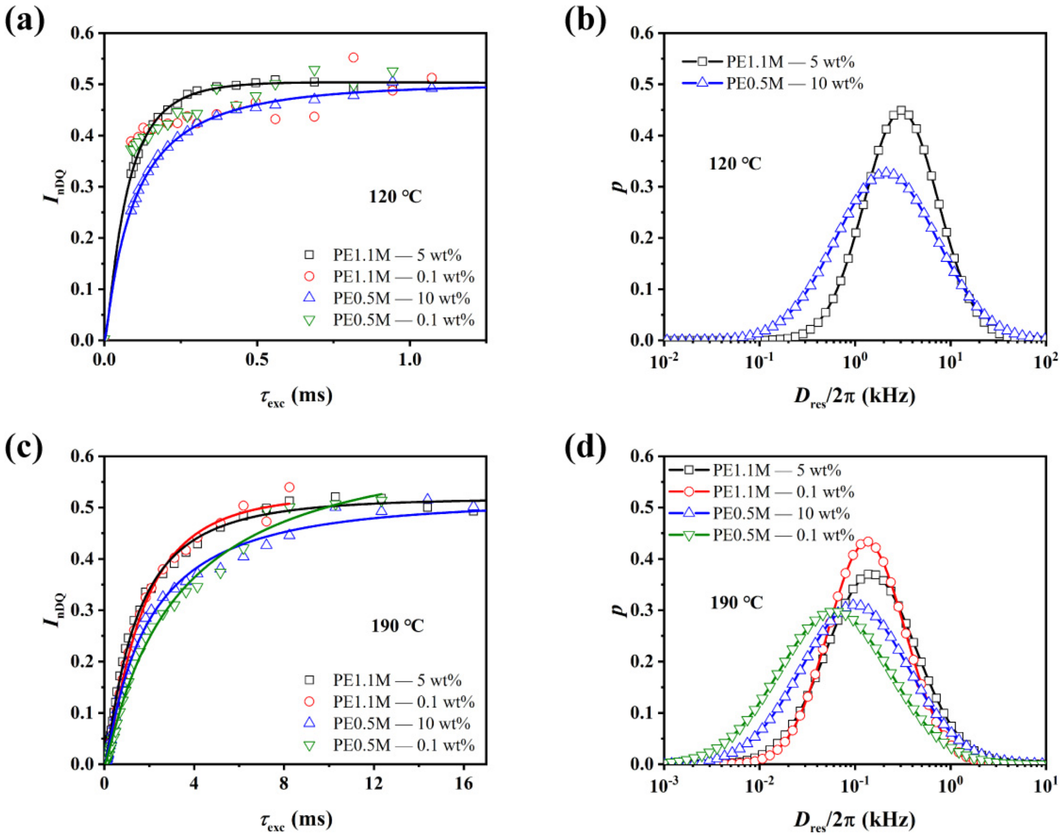

Figure S5. Comparison of nDQ buildup curves from all of the samples at (**a**) (**b**): 120 °C and (**c**) (**d**): 190 °C. The solid lines in (**a**) and (**c**) represent fitting curves of I_{nDQ}.

nDQ buildup curves of less-entangled samples at 120 °C could not be fitted with the above approach since their original DQ signals decay from the first sampling point. The higher D_{med} in Figure S5b reflected more constraints from crystalline lamellae and topological entanglements in PE1.1M—5 wt% than PE0.5M—10 wt%. In contrast, the growth rates of nDQ buildup curves at 190 °C were consistent with the initial entanglement density retained from a solution of all the PE samples due to a balance between chain relaxation and re-entanglement.
